# Intercropping with pear and cover crops as a strategy to boost soil carbon sequestration in the Taihang mountains’ fragile ecosystems

**DOI:** 10.3389/fpls.2025.1695802

**Published:** 2025-12-11

**Authors:** Chi Zhang, Shanshan Tong, Ruifang Zhang, Xuguang Li, Xin-Xin Wang, Hong Wang

**Affiliations:** 1 College of Land and Resources, Hebei Agricultural University, Baoding, China; 2 College of Resources and Environment Science, Hebei Agricultural University, Baoding, China; 3 National Research Center of Agricultural Engineering Technology in Northern Mountainous Areas, Hebei Agricultural University, Baoding, China; 4 Cultivated Land Quality Monitoring and Protection Center of Hebei Province, Shijiazhuang, China; 5 College of Horticulture, Hebei Agricultural University, Baoding, China

**Keywords:** fragile ecological zone, Taihang Mountains, pear orchard intercropping, land use, carbon stock

## Abstract

**Introduction:**

Soil carbon sequestration capacity in ecologically fragile areas of the Taihang Mountains’ gneiss slopes demands immediate attention. This study evaluated the synergistic effects of land-use transition (from barren hills to cropland to pear orchards) and cover crop intercropping on soil carbon storage.

**Methods:**

Field sampling and experiments were conducted at 72 sites in Fuping County. The analysis combined multi-index assessment of soil physicochemical properties with partial least squares structural equation modeling (PLS-SEM).

**Results:**

Land-use transition significantly increased soil carbon storage. In 8-year-old pear orchards, the organic carbon storage in the 0-20 cm soil layer reached 26.08 tC/ha, representing a 151.89% increase compared to cultivated land (10.35 tC/ha). Meanwhile, soil carbon storage in the 20-40 cm layer increased by 83.97% to 13.58 tC/ha. Under the cover cropping pattern, ryegrass in the surface 0-20 cm soil of 8-year-old orchards showed an 18.5% improvement in efficiency over natural grass. In the 20-40 cm deep soil, winter rape increased organic carbon content by 22.43% to 10.59 g/kg.

**Discussion:**

The synergistic mechanism was attributed to increased carbon input from cover crops and root systems, optimized soil physical structure (bulk density decreased by 5.1%-8.0%, porosity increased by 2.1%-4.1%), and formation of a nutrient-carbon pool synergy. Available potassium, phosphorus, and organic carbon were significantly positively correlated. This pear orchard and cover crop intercropping system exemplifies a practical Nature-based Solutions (NbS) pathway, achieving an annual carbon sink growth rate of 4.2-5.8 tC/(ha·a). Its core mechanism lies in constructing a triple synergy of “carbon sequestration enhancement, structural optimization, and nutrient cycling”, which enhances ecosystem services multidimensionally while fostering a resilient agricultural production system. This practice provides a technical paradigm for synergizing carbon neutrality goals and ecological restoration in fragile mountainous areas.

## 1 Introduction

Global warming poses a major threat to sustainable development, primarily driven by greenhouse gas emissions (Sokolov, 2021; Surya et al., 2023). As the world’s largest agricultural emitter, China has committed to carbon peak by 2030 and carbon neutrality by 2060 (IEA, 2023). However, agriculture accounts for about 14% of national emissions, presenting significant mitigation challenges (Jiang and Fu, 2021; Raza et al., 2023; Xia et al., 2023). Land use transition plays a crucial role in addressing these challenges. While energy and industrial activities dominate China’s emissions, land use change serves as a vital carbon sink - absorbing 1.315 billion tons of CO_2_ according to national reports. Strategies like returning farmland to forest effectively enhance carbon sequestration while supporting sustainable development (Shao et al., 2019). Critically, land use transition shapes regional carbon storage by modifying ecosystems, hydrology, and soil carbon dynamics, particularly influencing soil organic carbon distribution and sequestration processes (Yang et al., 2017; Lipatov et al., 2021; Yuan et al., 2021; Wang et al., 2024).

Under intensified human activities and climate warming, ecologically fragile regions such as the Taihang Mountains are confronting ecological crises including land desertification and vegetation degradation (Fan et al., 2021). This area features shallow soil layers and high susceptibility to water erosion, while its limestone parent material accelerates soil organic matter mineralization, creating a “carbon leakage” risk. Reduced vegetation coverage intensifies surface runoff erosion, leading to physical relocation of soil carbon pools and accelerated organic carbon decomposition through aggregate destruction (Wang et al., 2022). Although the Taihang Mountains possess substantial vegetation carbon sequestration potential, current monoculture plantations limit carbon sink functionality (Tilahun et al., 2022). Our research demonstrates that implementing pear orchard intercropping systems effectively stabilizes topsoil through root systems, increases labile carbon fractions by 18.5% via litter input, and alleviates nitrogen limitation through leguminous herbs’ biological nitrogen fixation, forming a dual carbon sequestration mechanism of “physical protection-biochemical regulation”. The validated “pear orchard + herbaceous cover” model enhances carbon storage in typical Taihang Mountain slopes by 4.2-5.8 tC/(hm²×a) annually, primarily through three mechanisms: extended green periods from herbs mitigating seasonal carbon sink gaps, synergistic root systems constructing carbon storage spaces, and enhanced plant diversity stimulating soil microbial carbon pumps (Sun et al., 2024). This approach provides crucial technical support for strengthening the ecological barrier functions of the Taihang Mountains within the dual carbon goals framework.

Land use transition significantly affects the rate and storage of organic carbon mineralization by impacting soil organic matter inputs, vegetation biomass, and physio-chemical properties (Economic Daily, 2020; Liu et al., 2024a). Current research utilizes field surveys (high but limited accuracy), model simulations (Yi et al., 2022), and remote sensing (focused on large-scale) (Dangulla et al., 2021; Lipatov et al., 2021), confirming significant variations in carbon storage responses across different land use methods (Totti et al., 2025). Ecologically fragile areas are particularly susceptible to degradation. Farmland contributes approximately half of agricultural carbon emissions (Liu et al., 2023), including 90% N_2_O, 70% CH_4_, and 20% of CO_2_ emissions (Li et al., 2023a, 2023b). Optimization models can enhance the carbon sequestration potential in these regions. Farmland carbon sequestration compensates between 0.1% and 27% of annual greenhouse gas emissions (Rodrigues et al., 2021). The combined ecological protection of cultivated land and surrounding areas sequesters approximately 9.60×10^6^ tons of carbon (Liu et al., 2024b), while the soil organic carbon stock in the top 0–40 cm of *Pinus tabuliformis* plantations reaches 8.7 kg/m² (Xu et al., 2022). In orchard systems, the expansion of pear orchards has contributed to an increase in storage capacity by 44.4 million tons, with organic carbon levels surpassing those of cultivated land (Duan et al., 2021). Converting farmland to forest has also enhanced the carbon pool (Yu et al., 2022). Cover crop management, including orchard grass and intercropping ryegrass in pear orchards, has increased the carbon pool by 21% and reduced the carbon footprint by 36.7% (Fu et al., 2023; He et al., 2023). Additionally, alfalfa cover cropping increased organic carbon by 14.3% within the 0–10 cm soil layer (Zhao et al., 2023). Meta-analyses indicate that cover planting can augment the soil carbon pool by approximately 31.1%, with a higher increase of 32.3% observed in mountainous regions compared to clear tillage systems (Li et al., 2023c).

This study involved field investigations and experiments on gneiss slopes of the Taihang Mountains to quantify the effects of intercropping cover crops in pear orchards on soil carbon storage. The research aimed to analyze the key factors driving synergistic carbon sequestration and identify optimal management practices, providing a scientific basis for sustainable land use and carbon neutrality policies.

## 2 Materials and methods

### 2.1 Overview of the research area

**Figure 1:**
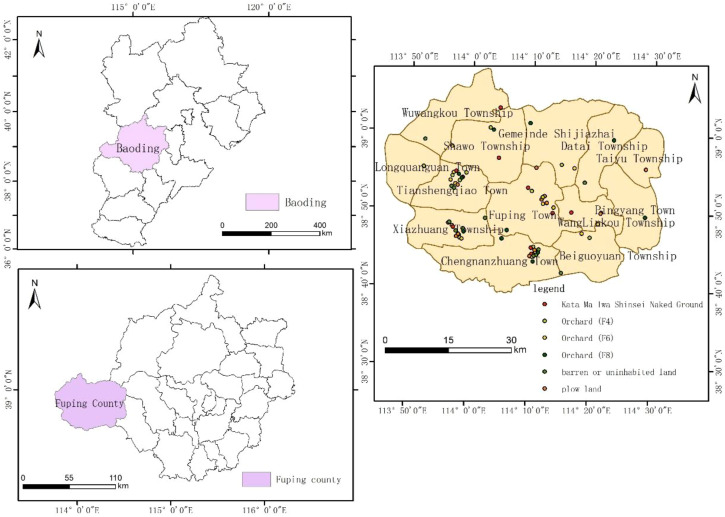
Location map of the research area.

The study area is located at an altitude of 302 m in the Agricultural Ecological Demonstration Park on Fuping Avenue, Baoding City, Hebei Province (115.48°E, 38.86°N) (**Figure 1**). Baoding City is situated on the eastern foothills of the northern Taihang Mountains. It has a temperate continental monsoon climate and is located in a warm temperate semi-humid area. Winters are characterized by cold, dry and mild snow, whereas summers are hot, humid and feature concentrated precipitation. The region experiences an average annual temperature of 12.6°C, with annual precipitation ranging from 550 to 790 mm, and a frost-free period lasting between 140 and 190 days.

**Figure 2:**
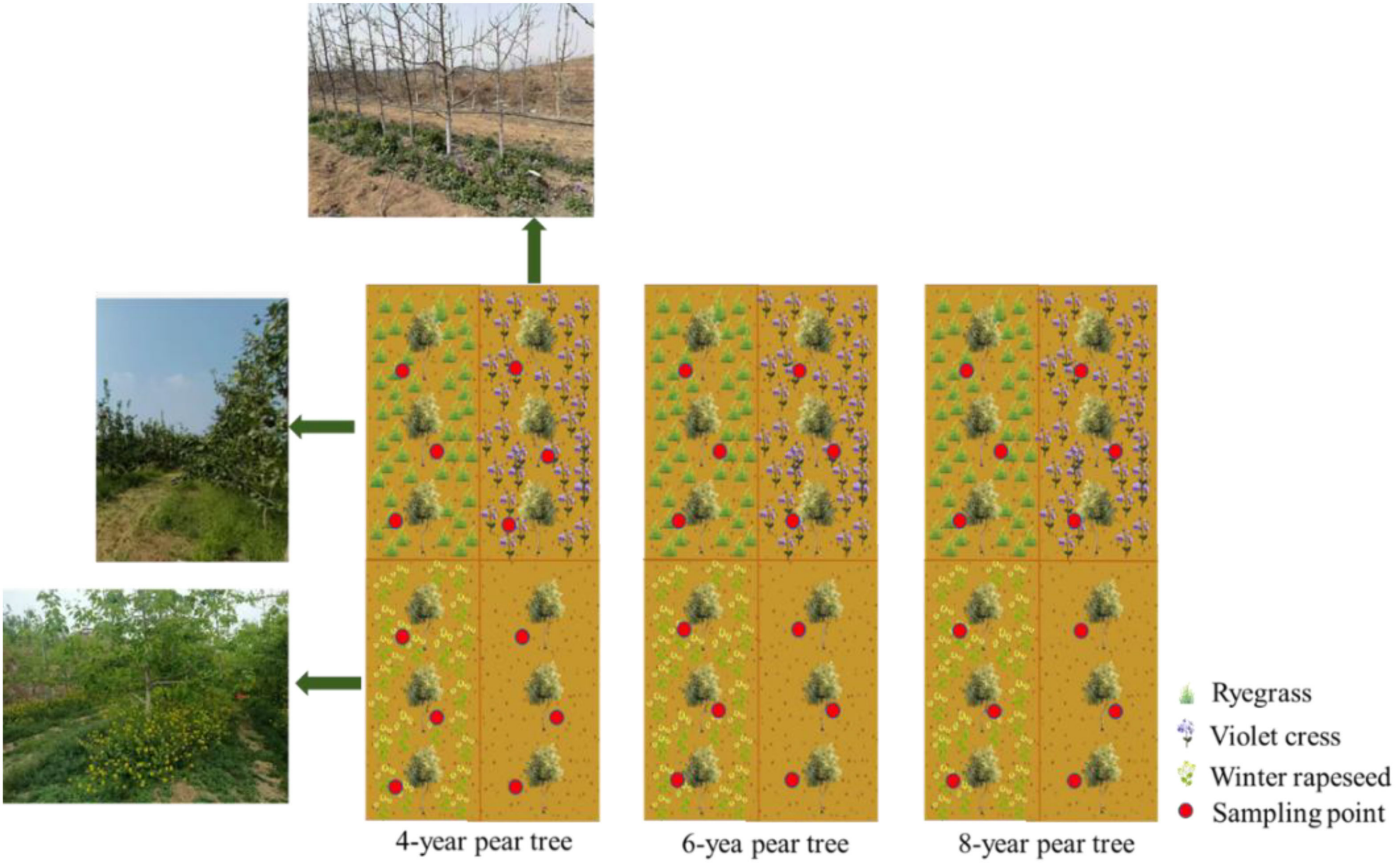
Experimental design layout diagram.

The land transformation project in Fuping County commenced in 2016, involving mechanical compaction of barren hillslopes and thorough mixing with weathered surface materials to construct terraced fields with slopes of approximately 30 degrees and widths exceeding 6 meters. This process resulted in the formation of a gneiss-derived soil layer approximately 60 cm thick. On this reconstructed substrate, the cultivation of maize and pear trees facilitated the conversion of barren hillslopes into cropland and orchards. Sampling sites for this study were selected from soils representing different transformation models, specifically encompassing four types: (1) newly formed soil bare land created by mechanically crushed gneiss bedrock in 2016; (2) cultivated land developed after seven years of maize planting; (3) orchard soils where Yuluxiang pear trees had been cultivated with fertilization for 4, 6, and 8 years (corresponding to tree ages in 2024); (4) combined planting systems integrating three cover crops (ryegrass, violet cress, and winter rapeseed) with pear trees of 4, 6, and 8 planting years, respectively. For each transformation model, 12 sampling points were established, totaling 72 sample points (**Figure 2**).

### 2.2 Experimental design

#### 2.2.1 Soil sampling sites and land use stages in the study area

In this study, soil sampling points were selected from gneiss barren hills and slopes, representing various land use stages after transformation. Specifically, samples included: (1) the original soil on barren hills and slopes; (2) newly formed soil from mechanically crushed gneiss bedrock (2016); (3) cultivated land formed after seven years of corn planting; (4) orchard soils where Yuluxiang pear trees had been planted and fertilized for 4, 6, and 8 years (corresponding to tree ages in 2024). The test crop was Yuluxiang pear, and the cover crops—ryegrass (R), violet cress (V), and winter rapeseed (W)—were supplied by the Hebei Academy of Agriculture and Forestry Sciences.

#### 2.2.2 Delineation of ecologically fragile areas

This study was conducted in the core experimental zone of Fuping County, Baoding City, Hebei Province, specifically focusing on the Agricultural Ecological Demonstration Park along Fuping Avenue and surrounding villages implementing ecological restoration projects. The area represents a typical low mountainous and hilly region in the northern section of the eastern Taihang Mountains. Using a GIS-based spatial analysis platform, we applied a comprehensive evaluation system integrating slope gradient, soil erosion intensity, vegetation coverage, and a lithology-soil depth composite index. This multi-criteria approach, combined with field validation from 72 sampling sites, enabled precise delineation of ecologically fragile slopes. The results demonstrate that these vulnerable areas are predominantly distributed on sunny and semi-sunny slopes at elevations of 300–500 meters with gradients of 10°-25°. These locations not only constitute ecologically sensitive zones but also provide suitable site conditions for establishing and developing pear orchard ecosystems.

#### 2.2.3 Experimental design for the impacts on soil carbon storage

Three cover crops, namely ryegrass, violet cress and winter rapeseed, were grown in a two-factor design and combined with pear trees with 4, 6 and 8 planting years, respectively. The pear trees were planted as control. A total of 12 treatments with three replicates of each were planted with 1.5 × 6 m distance and the area of each block was 54 m². On Apr 16, 2023, three mulching crops were planted between the rows of pear trees. Sampling of mature pear trees was conducted on Sep 2, 2024. Soil samples ranging from 0 to 40 cm were collected in layers for testing and analysis. The 0–40 cm soil profile was selected for layered sampling based on its ecological significance as the primary zone for biological activity and its demonstrated responsiveness to agricultural management interventions, providing optimal detection of carbon sequestration dynamics. Except for planting, the field management of each treatment remains consistent (**Figure 2**).

#### 2.2.4 Soil measurements

To comprehensively assess soil fertility and quality, determined a number of key soil indicators were selected which are:

1. Physical and chemical properties: includes soil organic matter, pH, total salinity, soil bulk density and soil porosity;
2. Nutrient elements: total nitrogen, available phosphorus and potassium;
3. Medium elements: exchangeable calcium, magnesium and available sulfur;
4. Trace elements: involving effective iron, manganese, copper and zinc.

### 2.3 Research methods

#### 2.3.1 Data acquisition

The soil sampling was carried out in accordance with the “Technical Regulations for Monitoring the Quality of Cultivated Land (NY/T1119-2012)” (National Agricultural Technology Extension and Service Center, 2012). A soil sample was collected using a 4 cm diameter spiral drill from the 0–40 cm depth, divided into two 20 cm layers. A 15-drill mixed sampling method was implemented between the pear tree grows in each test plot. The drilled soil was mixed evenly and the excess part was removed following the four-part method and packed into zip bags. Fifteen soil samples were simultaneously collected from the adjacent bare land and mixed evenly as the control. The samples were naturally air-dried and sieved by the 20 mm and 100 mm-mesh sieves to determine the soil nutrients. In addition, a 100 cm³ ring knife was used to collect soil to determine the soil bulk density of mature pear trees.

#### 2.3.2 Detection methods for sample indicators

The determination methods of various soil indicators refer to the book “Soil Agrochemical Analysis” (Bao, 2000):

1. Soil Organic Matter: Determined by the oil bath heating-potassium dichromate oxidation volumetric method. Precisely 0.1 g of air-dried soil sample passed through a 0.15 mm sieve was weighed into a hard glass tube. Then, 5.00 mL of 0.8 mol/L potassium dichromate (K_2_Cr_2_O_7_) standard solution and 5 mL of concentrated sulfuric acid (H_2_SO_4_) were added. The mixture was heated in an oil bath at 170–180 °C for 5 minutes. After cooling, the digest was transferred to a conical flask and titrated with a 0.2 mol/L ferrous sulfate (FeSO_4_) standard solution, using o-phenanthroline as an indicator. The organic carbon content was calculated based on the amount of ferrous sulfate consumed and then multiplied by the 1.724 coefficient to obtain the organic matter content.
2. pH: Measured potentiometrically. 10.00 g of air-dried soil passed through a 2 mm sieve was placed in a 50 mL beaker. 25 mL of CO_2_-free distilled water was added at a soil-to-water ratio of 1:2.5. The suspension was stirred for 1 minute and left to stand for 30 minutes. The supernatant was measured using a calibrated pH meter (Model PHSJ-4F), and the reading was recorded after stabilization, accurate to 0.01 pH unit.
3. Soil Bulk Density: Determined by the core method. A standard 100 cm³ core cutter was vertically pressed into undisturbed soil. After removal, both ends were trimmed flat. The soil core was dried to constant weight in an oven at 105 °C for 8 h. The mass of the dried soil sample was weighed, and the bulk density (g/cm³) was calculated based on the core cutter’s volume.
4. Total Nitrogen: Determined by the Kjeldahl digestion method. 1.000 g of soil sample passed through a 0.15 mm sieve was placed in a digestion tube. 1.8 g of accelerator (a 10:1 mixture of potassium sulfate, K_2_SO_4_, and copper sulfate, CuSO_4_) and 5 mL of concentrated sulfuric acid (H_2_SO_4_) were added. Digestion was performed at 420 °C for 2 h until the solution turned bluish-green. After cooling, distillation was carried out using a Kjeldahl nitrogen analyzer (Model K9860). The distillate was absorbed in a 2% boric acid (H_3_BO_3_) solution and titrated with 0.01 mol/L hydrochloric acid (HCl) standard solution, using a methyl red-bromocresol green mixed indicator for endpoint determination.
5. Available Phosphorus: Determined by sodium bicarbonate extraction-molybdenum antimony ascorbic acid colorimetry. 2.50 g of soil sample was added to 50 mL of 0.5 mol/L sodium bicarbonate (NaHCO_3_) extraction solution (pH 8.5). The mixture was shaken for 30 minutes and then filtered. 5 mL of the filtrate was mixed with 5 mL of molybdenum-antimony-ascorbic acid color developing reagent. After developing color at room temperature for 30 minutes, the absorbance was measured at 880 nm wavelength using a UV-Vis spectrophotometer (Model UV-1800). The concentration was calculated via a standard curve.
6. Available Potassium: Determined by ammonium acetate extraction-flame photometry. 5.00 g of soil sample was added to 50 mL of 1 mol/L ammonium acetate (CH_3_COONH_4_) solution (pH 7.0). The mixture was shaken for 15 minutes and then filtered. The filtrate was analyzed using a flame photometer (Model FP6410) with a potassium filter at a wavelength of 768 nm. Quantification was performed using a standard curve prepared from a series of potassium standard solutions.
7. Exchangeable Calcium and Magnesium: Determined by ammonium acetate exchange-atomic absorption spectrophotometry. 10.00 g of soil sample was sequentially extracted three times using a total of 50 mL of 1 mol/L ammonium acetate (CH_3_COONH_4_) solution (pH 7.0). The combined filtrates were analyzed using an atomic absorption spectrophotometer (Model AA-6880). The wavelengths were set at 422.7 nm for calcium and 285.2 nm for magnesium, with deuterium lamp background correction.
8. Available Sulfur: Determined by phosphate-acetic acid extraction-barium sulfate turbidimetry. 10.00 g of soil sample was added to 50 mL of extraction solution (0.016 mol/L calcium phosphate, Ca(H_2_PO_4_)_2_, + 2 mol/L acetic acid, CH_3_COOH). The mixture was shaken for 1 hour and then filtered. 10 mL of the filtrate was mixed with 1 mL of stabilizer (glycerol:ethanol = 1:1) and 0.5 g of barium chloride (BaCl_2_) crystals. After shaking for 1 minute, the turbidity was measured at 440 nm wavelength using a spectrophotometer.
9. Available Trace Elements (Fe, Mn, Cu, Zn): Determined by DTPA extraction-atomic absorption spectrophotometry. 20.00 g of soil sample was added to 40 mL of DTPA extraction solution (0.005 mol/L DTPA + 0.01 mol/L calcium chloride, CaCl_2_ + 0.1 mol/L triethanolamine, TEA, pH 7.3). The mixture was shaken for 2 h and then filtered. The filtrate was analyzed using an atomic absorption spectrophotometer at the following wavelengths: Fe 248.3 nm, Mn 279.5 nm, Cu 324.8 nm, Zn 213.9 nm.

All determinations included blank controls and quality control using certified reference material (GSS-17). Each sample was analyzed in triplicate.

#### 2.3.3 Data analysis

Excel 2019 and SPSS Statistics 26 software were used to organize and statistically analyze the experimental data. The effects of land use patterns, planting years, and their interactions on soil organic carbon content and carbon storage were quantitatively assessed. Correlation analysis was conducted using the Pearson correlation coefficient method. The Shapiro-Wilk test was applied to all continuous variables to assess normality. For variables meeting the normality assumption, Pearson correlation analysis was employed; For those violating normality, the more robust Spearman’s rank correlation analysis was used instead. The partial least squares structural equation modeling (PLS-SEM) was constructed and analyzed using R 4.5.1 software.

## 3 Results

### 3.1 Soil chemical properties

The soil nutrient indicators of the newly formed gneiss bare land are higher than those of the original gneiss hillside soil. Specifically, organic carbon, total nitrogen, available phosphorus, potassium, exchangeable calcium, magnesium, sulfur, effective manganese and zinc in the newly formed bare land were significantly higher than hillside soil. Among them, the differences between available phosphorus and potassium are obvious. The pH values of the both soils are alkaline, but the difference were non-significant. There was no significant difference in the contents of effective iron and copper between both soil types. This data provides basic soil background information for the subsequent analysis (**Table 1**).

**Table 1:** Soil chemical properties.

Soil at the sampling site	Gneiss hillside soil	Gneiss is a bare ground of new soil
Organic carbon (g/kg)	3.20 ± 0.40	3.92 ± 0.89
Total nitrogen (g/kg)	0.31 ± 0.04	0.38 ± 0.03
Available phosphorus (mg/kg)	4.31 ± 0.75	13.52 ± 2.04
Available potassium (mg/kg)	46.22 ± 8.65	77.15 ± 9.84
pH	7.70 ± 0.26	7.55 ± 0.04
Exchangeable calcium (mg/kg)	726.0 ± 168.31	930.7 ± 137.89
Exchangeable magnesium (mg/kg)	179.67 ± 19.86	234.67 ± 30.57
Effective sulfur (mg/kg) Effective sulfur	22.97 ± 3.56	25.57 ± 5.11
Effective iron (mg/kg)	11.80 ± 2.57	12.33 ± 1.94
Effective manganese (mg/kg)	2.50 ± 0.26	3.60 ± 0.50
Effective copper (mg/kg)	0.60 ± 0.05	0.66 ± 0.17
Effective zinc (mg/kg)	3.20 ± 0.40	3.92 ± 0.89

### 3.2 Effects of different land use patterns on soil carbon sequestration capacity

Land use transition markedly impacted soil organic carbon (SOC) content and storage. In the topsoil (0–20 cm), crop land (P) had significantly higher SOC level than bare land (G), with a of 55.4% increase. Additionally, 4-year (F4), 6-year (F6), and 8-year (F8) pear orchards showed SOC increases of 80.5%, 103.6%, and 138.0%, respectively, compared to cropland. In the subsoil (20–40 cm), SOC content in the 8-year orchards was significantly higher (200.3%) than cropland. For carbon storage, topsoil carbon storage in pear orchards increased significantly by 114.7% compared to cropland. The carbon storage in these orchards was increased in F4, F6, and F8 by 296.7%, 373.4%, and 441.1%, respectively. Subsoil carbon storage also showed upward trend, with the 8-year orchards storing 418.3% more than cropland. Regarding soil layer differences: The SOC storage of the 0–20 cm topsoil in all treatments was significantly higher than 20–40 cm subsoil (**Table 2**). Overall, following the transition from cropland to pear orchards, SOC content and storage demonstrated a continuous and positive trend with longer cultivation duration, particularly in the topsoil. This indicates that among various land use transition models, pear orchards maximize the soil carbon sequestration capacity which enables an ideal land use pattern for optimizing regional carbon cycling and strengthening the soil carbon sink.

**Table 2:** Carbon stocks in different soil layers under different land uses.

Treatment	Soil layers (cm)	Soil organic carbon (g/kg)	Soil organic carbon stocks (tC/hm^2^)
G	0-20	6.16 ± 2.41aD	4.82 ± 1.09aE
	20-40	2.88 ± 0.69bC	2.62 ± 0.67aD
P	0-20	9.57 ± 0.94aC	10.35 ± 0.90aD
	20-40	5.77 ± 1.09bABC	7.38 ± 1.45bC
F4	0-20	11.12 ± 0.26aBC	19.12 ± 0.96aC
	20-40	5.62 ± 0.42bBC	9.17 ± 0.77bBC
F6	0-20	12.54 ± 0.75aAB	22.82 ± 1.34aB
	20-40	7.43 ± 0.79bAB	11.77 ± 1.11bAB
F8	0-20	14.66 ± 0.29aA	26.08 ± 0.57aA
	20-40	8.65 ± 0.16bA	13.58 ± 0.50bA

Different lowercase letters indicate statistically significant differences (*P<*0.05) between soil layers within the same treatment, while different uppercase letters indicate significant differences (*P<*0.05) among treatments within the same soil layer. Data are presented as mean ± standard deviation (n=3). G: Bare land; P: Crop land; F4: Pear-herb intercropping system (4-year trees); F6: Pear-herb intercropping system (6-year trees); F8: Pear-herb intercropping system (8-year trees).

### 3.3 Effects of pear-cover crops mixed cropping on soil carbon sequestration capacity

The practical application effect of the pear-herb mixed cropping model in this region will be comprehensively evaluated. In order to provide a scientific basis for regional ecological restoration and sustainable agricultural development.

#### 3.3.1 Soil macronutrient variations in relation to pear orchard age and cover crop

Soil nutrients in pear orchards increased significantly with stand age (P<0.05). In the 0–20 cm topsoil, 8-year-old orchards showed the most pronounced increases relative to 4-year-old stands: organic matter (+31.8%), available phosphorus (+59.4-64.6%), and available potassium (+76.6-92.6%). Intercropping with ryegrass and winter rapeseed further enhanced these age-related gains. Ryegrass particularly improved organic matter (+28.1%) and total nitrogen (+19.8%), whereas winter rapeseed most effectively boosted available potassium (**Table 3**). Although nutrient levels were generally lower in the 20–40 cm subsoil, both orchard age and cover crops still exerted significant effects (*P* < 0.05). Eight-year-old orchards exhibited increases in organic matter (+53.9-58.7%), available phosphorus (+26.2-30.8%), and available potassium (+61.9-97.5%). Winter rapeseed was particularly effective in enhancing subsoil potassium (+54.9%), though the magnitude of phosphorus increase in the subsoil was lower than that observed in the topsoil (**Table 3**).

**Table 3:** Changes in soil macronutrients at different pear ages and cover crop treatments.

Soil traits	Year	Cover crops	0-20 (cm)	20-40 (cm)
Total porosity (%)	4	N	47.48 ± 1.95a	43.02 ± 0.63a
		R	50.37 ± 1.67a	44.84 ± 0.81a
		W	51.27 ± 1.26a	45.96 ± 0.93a
		V	50.27 ± 2a	45.71 ± 1.02a
	6	N	47.31 ± 2.89a	44.37 ± 2.08a
		R	50.26 ± 1.36a	46.06 ± 0.75a
		W	50.4 ± 1.31a	46.67 ± 1.1a
		V	50.58 ± 2.64a	46.59 ± 2.55a
	8	N	49.98 ± 1.47b	45.11 ± 1.02a
		R	52.03 ± 2.8a	46.45 ± 1.73a
		W	51.55 ± 3.85a	47.82 ± 0.33a
		V	51.33 ± 1.87a	47.15 ± 1.07a
pH	4	N	7.94 ± 0.23a	8.47 ± 0.08a
		R	7.87 ± 0.08a	7.83 ± 0.26b
		W	7.84 ± 0.27a	7.95 ± 0.22ab
		V	7.87 ± 0.17a	7.81 ± 0.08b
	6	N	8.1 ± 0.1a	8.04 ± 0.14a
		R	7.94 ± 0.26a	8.3 ± 0.07a
		W	7.74 ± 0.3a	7.98 ± 0.41a
		V	7.84 ± 0.09a	8.25 ± 0.06a
	8	N	7.93 ± 0.15a	8.15 ± 0.18a
		R	8.01 ± 0.06a	8.25 ± 0.04a
		W	7.74 ± 0.3a	8.09 ± 0.16a
		V	8.08 ± 0.09a	7.71 ± 0.34a
AK (mg/kg)	4	N	192.37 ± 6.09b	70.88 ± 7.44a
		R	225.92 ± 5.83ab	77.43 ± 7.13a
		W	233.87 ± 2.89a	90.36 ± 4.03a
		V	200.77 ± 25.06ab	87.58 ± 13.71a
	6	N	244.6 ± 7.78b	95.32 ± 10.54a
		R	286.81 ± 8.15a	117.09 ± 8.36a
		W	284.89 ± 9.3ab	119.27 ± 14.08a
		V	266.21 ± 27.56ab	105.59 ± 2.64a
	8	N	339.66 ± 5.94b	114.84 ± 3.01a
		R	389.4 ± 7.7a	133.2 ± 8.63b
		W	385.22 ± 19.59a	139.98 ± 4.52a
		V	373.97 ± 15.58ab	132.22 ± 6.76ab
AP (mg/kg)	4	N	100.83 ± 6.83a	47.45 ± 1.99a
		R	128.28 ± 10.76a	51.03 ± 5.72a
		W	128.46 ± 1.85a	51.75 ± 5.6a
		V	122.11 ± 10.54a	50.07 ± 2.54a
	6	N	148.9 ± 6.89b	51.78 ± 6.88a
		R	185.46 ± 3.71a	53.86 ± 3.9a
		W	183.99 ± 9.44a	56.91 ± 3.92a
		V	161.63 ± 17.64ab	53.84 ± 2.82a
	8	N	160.65 ± 10.42b	59.88 ± 1.06a
		R	201.53 ± 15.01a	61.86 ± 6.83a
		W	200.37 ± 2.76a	62.01 ± 3.89a
		V	186 ± 14.12aab	62.8 ± 6.01a
SOM (g/kg)	4	N	19.17 ± 0.45b	9.69 ± 0.72a
		R	22.75 ± 0.86a	10.66 ± 0.92a
		W	22.75 ± 0.6a	11.51 ± 1.66a
		V	21.39 ± 1.1ab	10.6 ± 1.27a
	6	N	21.61 ± 1.29b	12.81 ± 1.37a
		R	25.44 ± 1.75a	13.61 ± 0.98a
		W	25.16 ± 1.67ab	14.04 ± 0.57a
		V	24.83 ± 2.45ab	13.76 ± 1.8a
	8	N	25.27 ± 0.5b	14.91 ± 0.27b
		R	29.15 ± 0.36a	17.4 ± 1.16ab
		W	27.49 ± 0.83ab	18.26 ± 0.13a
		V	26.38 ± 0.96ab	17.67 ± 1.13ab
Total nitrogen (g/kg)	4	N	1.05 ± 0.02a	0.57 ± 0.07a
		R	1.21 ± 0.06a	0.59 ± 0.04a
		W	1.21 ± 0.03a	0.61 ± 0.05a
		V	1.16 ± 0.08a	0.59 ± 0.01a
	6	N	1.11 ± 0.02b	0.6 ± 0.01a
		R	1.29 ± 0.1a	0.63 ± 0.1a
		W	1.21 ± 0.07ab	0.64 ± 0.03a
		V	1.18 ± 0.08ab	0.62 ± 0.04a
	8	N	1.27 ± 0.02b	0.68 ± 0.07a
		R	1.45 ± 0.03a	0.78 ± 0.02a
		W	1.38 ± 0.07ab	0.77 ± 0.03a
		V	1.31 ± 0.05ab	0.74 ± 0.06a

Different lowercase letters within the same column for each soil depth and year indicate statistically significant differences among cover crop treatments (*P* < 0.05). Values sharing the same letter are not significantly different. Data are presented as mean ± standard deviation (n=3). Abbreviations for cover crop treatments: N, No cover crop (control); R, Ryegrass; W, Winter rapeseed; V, Violet cress.

#### 3.3.2 Impact of tree maturity and cover cropping on soil trace element composition

In 8-year-old pear orchards, exchangeable calcium and magnesium increased by 33.9%-61.6% and 24.0%-46.3% respectively, while available sulfur rose by 88.8%-128.1% compared to 4-year-old orchards. Ryegrass treatment performed optimally, achieving exchangeable calcium and magnesium levels of 2095 mg/kg and 446.33 mg/kg respectively in 8-year-old orchards, with calcium content 20.8% higher than natural fallow. Compared to nitrogen-only treatment, ryegrass intercropping enhanced exchangeable calcium and magnesium by 25.5% and 19.5% in 6-year-old orchards, and by 30.3% and 20.8% in 8-year-old orchards. In the 20–40 cm subsoil, 8-year-old orchards reached exchangeable calcium levels of 2117.67-2328.33 mg/kg (45.4%-59.9% increase), with magnesium increasing by 57.8%-81.8% and available sulfur by 148.6%-183.5%, showing the most effectiveness under winter rapeseed treatment (**Table 4**). Although cover crops had weaker ameliorative effects on the subsoil than on the topsoil, both ryegrass and winter rapeseed maintained relatively high nutrient levels.

**Table 4:** Changes in soil intermediate elements at different pear years and cover crop treatments.

Soil traits	Year	Cover crops	0-20 (cm)	20-40 (cm)
Exchanged Ca (mg/kg)	4	N	1296 ± 150.1a	1456.33 ± 201.75a
		R	1463 ± 85.19a	1658.33 ± 69.07a
		W	1496.67 ± 132.28a	1695.33 ± 165.41a
		V	1430 ± 223.58a	1515 ± 270.79a
	6	N	1423.33 ± 105.82b	2008.33 ± 97.2a
		R	1785.67 ± 41.53a	2197 ± 173.65a
		W	1763 ± 104.23ab	2230 ± 154.31a
		V	1614.67 ± 100.08ab	2139 ± 148.19a
	8	N	1734.67 ± 19.97b	2117.67 ± 140.28a
		R	2095 ± 162.25a	2328.33 ± 209.23a
		W	2022.33 ± 128.46ab	2328.33 ± 325.17a
		V	2004 ± 78.23ab	2139.33 ± 66.13a
Exchanged Mg (mg/kg)	4	N	305 ± 3.06a	241.67 ± 23.55a
		R	326 ± 11.36a	256.33 ± 28.29a
		W	328 ± 21.03a	251.33 ± 23.95a
		V	308 ± 19.09a	254.33 ± 27.48a
	6	N	340 ± 18.5b	303.33 ± 24.59a
		R	406.33 ± 8.65a	321.33 ± 11.17a
		W	402 ± 24.5ab	318 ± 20.01a
		V	379.67 ± 31.96ab	315.33 ± 14.33a
	8	N	378.33 ± 24.18b	381.33 ± 12.02a
		R	446.33 ± 24.63a	431.33 ± 10.71a
		W	434.67 ± 31.67ab	438.67 ± 15.9a
		V	434.67 ± 29.72ab	404.33 ± 27.03a
Effective S (mg/kg)	4	N	34.8 ± 6.98a	21.27 ± 1.68a
		R	42.37 ± 2.77a	22.9 ± 1.48a
		W	40.37 ± 3.12a	23.47 ± 1.06a
		V	39.03 ± 5.26a	22.97 ± 1.7a
	6	N	46.07 ± 0.69b	43.2 ± 2.66a
		R	60 ± 0.45a	48.27 ± 0.9a
		W	58.9 ± 5.6ab	48.5 ± 4.24a
		V	58.43 ± 5.37ab	47.7 ± 4.13a
	8	N	65.7 ± 4.57b	52.87 ± 3.77b
		H	79.37 ± 7.86a	57.17 ± 2.53ab
		W	77.97 ± 1.04ab	60.3 ± 0.44a
		V	76.57 ± 4.62ab	57.67 ± 1.33ab

Different lowercase letters within the same column for each soil depth and year indicate statistically significant differences among cover crop treatments (*P<*0.05). Values sharing the same letter are not significantly different. Data are presented as mean ± standard deviation (n=3). Abbreviations for cover crop treatments: N, No cover crop (control); R, Ryegrass; W, Winter rapeseed; V, Violet cress.

#### 3.3.3 Soil microelement fluctuations under different pear orchard ages and cover crop

Pear orchard age and cover crop management significantly enhanced trace element concentrations in the 0–20 cm topsoil (*P* < 0.05). Compared to 4-year-old orchards, 8-year-old orchards showed increases of 73.1%-110.0% in available Fe, 33.2%-58.6% in Mn, 45.5%-73.3% in Cu, and 114.5%-141.8% in Zn. Ryegrass treatment notably improved Fe and Mn levels, with available Mn reaching 7.50 mg/kg, whereas winter rapeseed was most effective in enhancing Zn, achieving 8.27 mg/kg—a 12.8% increase over nitrogen-only fertilization (**Table 5**). Although trace element concentrations in the 20–40 cm subsoil were lower than in the topsoil, they still increased significantly with orchard age (*P* < 0.05). In 8-year-old orchards, available Fe, Mn, Cu, and Zn rose by 42.8%-54.4%, 85.3%-103.9%, 67.0%-86.4%, and 193.4%-222.6%, respectively. Winter rapeseed treatment generally maintained higher levels of most trace elements in the subsoil (**Table 5**).

**Table 5:** Changes in soil microelements at different pear years and cover crop treatments.

Soil traits	Year	Cover crops	0-20 (cm)	20-40 (cm)
Effective Fe (mg/kg)	4	N	21.53 ± 1.32b	16.6 ± 1.31a
		R	26.93 ± 1.52a	18.43 ± 0.5a
		W	23.37 ± 1.07ab	18.67 ± 2.74a
		V	23.97 ± 2.48ab	17.2 ± 1.66a
	6	N	28.93 ± 2.32b	21.8 ± 1.18a
		R	35.9 ± 0.56a	22.9 ± 1.51a
		W	34.57 ± 1.92a	23.33 ± 1.42a
		V	35.63 ± 1.51a	22.6 ± 0.98a
	8	N	37.27 ± 2.32c	23.7 ± 2.07a
		R	45.2 ± 0.5a	25.63 ± 2.09a
		W	42.7 ± 1.33ab	25.57 ± 1.27a
		V	40.33 ± 0.74bc	24.6 ± 1.94a
Effective Mn (mg/kg)	4	N	4.73 ± 0.23a	3.53 ± 0.38a
		R	5.47 ± 0.28a	3.73 ± 0.41a
		W	4.93 ± 0.44a	3.7 ± 0.38a
		V	5.47 ± 0.38a	3.67 ± 0.26a
	6	N	5.77 ± 0.24b	4.07 ± 0.15a
		R	6.87 ± 0.52a	4.43 ± 0.37a
		W	6.6 ± 0.61a	4.5 ± 0.23a
		V	6.6 ± 0.36a	4.3 ± 0.35a
	8	N	6.3 ± 0.21b	6.57 ± 0.58a
		R	7.5 ± 0.35a	6.53 ± 0.45a
		W	7.13 ± 0.5ab	7.2 ± 0.69a
		V	6.97 ± 0.47ab	6.93 ± 0.65a
Effective Cu (mg/kg)	4	N	1.01 ± 0.06a	0.88 ± 0.03a
		R	1.18 ± 0.08a	1.01 ± 0.06a
		W	1.18 ± 0.08a	1.03 ± 0.14a
		V	1.18 ± 0.08a	1.04 ± 0.11a
	6	N	1.26 ± 0.08b	1.26 ± 0.17a
		R	1.56 ± 0.16a	1.26 ± 0.11a
		W	1.58 ± 0.05a	1.35 ± 0.09a
		V	1.56 ± 0.07a	1.29 ± 0.19a
	8	N	1.47 ± 0.06b	1.47 ± 0.11a
		R	1.75 ± 0.09a	1.51 ± 0.25a
		W	1.75 ± 0.13a	1.64 ± 0.05a
		V	1.62 ± 0.08ab	1.57 ± 0.07a
Effective Zn (mg/kg)	4	N	3.42 ± 0.23a	1.36 ± 0.02a
		R	3.93 ± 0.16a	1.47 ± 0.05a
		W	3.79 ± 0.29a	1.48 ± 0.25a
		V	3.59 ± 0.37a	1.41 ± 0.25a
	6	N	5.22 ± 0.07b	2.7 ± 0.21a
		R	6.44 ± 0.46a	3.04 ± 0.42a
		W	6.22 ± 0.53a	3.02 ± 0.09a
		V	6.45 ± 0.3a	2.97 ± 0.08a
	8	N	7.33 ± 0.16b	3.99 ± 0.06a
		R	8.08 ± 0.46ab	4.38 ± 0.2a
		W	8.27 ± 0.28a	4.39 ± 0.29a
		V	7.92 ± 0.14ab	4.37 ± 0.25a

Different lowercase letters within the same column for each soil depth and year indicate statistically significant differences among cover crop treatments (*P<*0.05). Values sharing the same letter are not significantly different. Data are presented as mean ± standard deviation (n=3). Abbreviations for cover crop treatments: N, No cover crop (control); R, Ryegrass; W, Winter rapeseed; V, Violet cress.

#### 3.3.4 Soil carbon storage variations under different pear orchard ages and cover crop

This study showed that pear orchard age and cover crop management had a significant effect on soil organic carbon content and carbon storage (*P<*0.05). Compared with natural grass fallow, ryegrass, rapeseed and violet cress treatments significantly increased soil organic carbon content and carbon storage in 0–20 cm surface soil. Compared with natural grass fallow, the organic carbon content of 4-year, 6-year and 8-year orchards increased by 18.7%, 17.6% and 15.4%, and the carbon storage increased by 24.4%, 20.3% and 18.5%, respectively. Winter rapeseed treatment was the second most effective to enhance the organic carbon content by 18.7%, 16.4% and 8.8% in 4-year, 6-year and 8-year orchards, and carbon storage by 24.1%, 17.1% and 15.0%, respectively (**Table 6**).

**Table 6:** Changes in soil organic carbon and carbon storage at different pear ages and cover crop treatments.

Soil traits	Year	Cover crops	0-20 (cm)	20-40 (cm)
SOC (g/kg)	4	N	11.12 ± 0.26b	5.62 ± 0.42a
		R	13.2 ± 0.5b	6.18 ± 0.53a
		W	13.2 ± 0.35a	6.68 ± 0.97a
		V	12.41 ± 0.64b	6.15 ± 0.74a
	6	N	12.54 ± 0.75b	7.43 ± 0.79a
		R	14.75 ± 1.01a	7.9 ± 0.57a
		W	14.59 ± 0.97b	8.14 ± 0.33a
		V	14.4 ± 1.42ab	7.98 ± 1.05a
	8	N	14.66 ± 0.29a	8.65 ± 0.16b
		R	16.91 ± 0.21b	10.09 ± 0.67ab
		W	15.95 ± 0.48ab	10.59 ± 0.08a
		V	15.3 ± 0.56a	10.25 ± 0.65ab
TSOC (tC/hm^2^)	4	N	19.12 ± 0.96b	9.17 ± 0.77a
		R	23.79 ± 0.48a	9.73 ± 0.74a
		W	23.74 ± 0.8ab	10.36 ± 1.64a
		V	22.86 ± 2.05ab	9.57 ± 1.24a
	6	N	22.82 ± 1.34b	11.77 ± 1.11a
		R	27.45 ± 0.81a	12.19 ± 0.94a
		W	26.73 ± 2.01ab	12.4 ± 0.25a
		V	26.92 ± 2.57ab	12.04 ± 1.09a
	8	N	26.08 ± 0.57b	13.58 ± 0.29a
		R	30.9 ± 1.73a	15.48 ± 1.24a
		W	29.99 ± 2.3ab	15.81 ± 0.2a
		V	29.1 ± 1.07ab	15.51 ± 1.15a

Different lowercase letters within the same column for each soil depth and year indicate statistically significant differences among cover crop treatments (*P<*0.05). Values sharing the same letter are not significantly different. Data are presented as mean ± standard deviation (n=3). Abbreviations for cover crop treatments: N, No cover crop (control); R, Ryegrass; W, Winter rapeseed; V, Violet cress.

Violet cress treatment showed significant improvement in the first 6 years, with an 11.6%-14.8% increase in organic carbon, whereas, increase was only 4.4% in 8-year orchards. Although the organic carbon content and carbon storage in the 20–40 cm subsoil increased with orchard age and no significant differences were observed with the change of cover crop. It is worth noting that compared with the 4-year orchard, the 8-year orchard showed a significant increase of 31.8%-52.1% and a 36.4%-61.6% in the organic carbon content and carbon storage in the surface soil (**Table 6**).

#### 3.3.5 Soil structure variations in relation to orchard age and cover crop treatments

Pear orchard age and cover crop management significantly improved the physical structure of the 0–20 cm topsoil. All cover crop treatments significantly reduced soil bulk density, with decreases of 5.1%-6.6%, 5.8%-8.0%, and 3.1%-3.8% in 4-year, 6-year, and 8-year-old orchards, respectively. Winter rapeseed treatment reduced bulk density to 1.27 and 1.29 g/cm³ in 4-year and 6-year-old orchards. Cover crops also significantly increased total soil porosity. In 8-year-old orchards, porosity under ryegrass, winter rapeseed, and violet cress reached 52.03%, 51.55%, and 51.33%, respectively, representing a 2.1%-4.1% increase over natural vegetation (49.98%). Soil porosity showed an increasing trend with orchard age, with ryegrass treatment achieving a 3.3% increase (**Table 7**). Although the physical properties of the 20–40 cm subsoil are influenced by the pear orchard age and the cover crop, the magnitude of the change is less pronounced than topsoil. Cover crop treatment, generally resulted in lower bulk density compared to natural vegetation, with the lowest value of 1.36 g/cm³ observed in 8-year orchards under winter rapeseed. The reductions in bulk density across 4-year, 6-year, and 8-year orchards ranged from 3.4%-5.4%, 3.4%-4.1%, and 2.8%-4.9%, respectively, Porosity increased by 1.4%-4.3% under cover crops relative to natural vegetation cover, though difference were not statistically significant (*P*>0.05). Notably, the 8-year orchard with winter rape exhibited the highest porosity of 47.82%, indicating that long-term orchard cultivation may promote subsoil structure (**Table 7**).

**Table 7:** Changes in soil physicochemical properties at different pear ages and cover crop treatments.

Soil traits	Year	Cover crops	0-20 (cm)	20-40 (cm)
Bulk density (g/cm^3^)	4	N	1.36 ± 0.05a	1.48 ± 0.02a
		R	1.29 ± 0.05a	1.43 ± 0.02a
		W	1.27 ± 0.03a	1.4 ± 0.02a
		V	1.29 ± 0.05a	1.41 ± 0.03a
	6	N	1.37 ± 0.08a	1.45 ± 0.05a
		R	1.29 ± 0.04a	1.4 ± 0.02a
		W	1.29 ± 0.04a	1.39 ± 0.03a
		V	1.28 ± 0.07a	1.39 ± 0.07a
	8	N	1.3 ± 0.04a	1.43 ± 0.03a
		R	1.25 ± 0.07b	1.39 ± 0.04a
		W	1.26 ± 0.1b	1.36 ± 0.01a
		V	1.27 ± 0.05ab	1.37 ± 0.03a
Total porosity (%)	4	N	47.48 ± 1.95a	43.02 ± 0.63a
		R	50.37 ± 1.67a	44.84 ± 0.81a
		W	51.27 ± 1.26a	45.96 ± 0.93a
		V	50.27 ± 2a	45.71 ± 1.02a
	6	N	47.31 ± 2.89a	44.37 ± 2.08a
		R	50.26 ± 1.36a	46.06 ± 0.75a
		W	50.4 ± 1.31a	46.67 ± 1.1a
		V	50.58 ± 2.64a	46.59 ± 2.55a
	8	N	49.98 ± 1.47b	45.11 ± 1.02a
		R	52.03 ± 2.8a	46.45 ± 1.73a
		W	51.55 ± 3.85a	47.82 ± 0.33a
		V	51.33 ± 1.87a	47.15 ± 1.07a

Different lowercase letters within the same column for each soil depth and year indicate statistically significant differences among cover crop treatments (*P<*0.05). Values sharing the same letter are not significantly different. Data are presented as mean ± standard deviation (n=3). Abbreviations for cover crop treatments: N, No cover crop (control); R, Ryegrass; W, Winter rapeseed; V, Violet cress.

### 3.4 Pathway analysis of pear and cover crop intercropping effects on soil carbon dynamics

**Figure 3:**
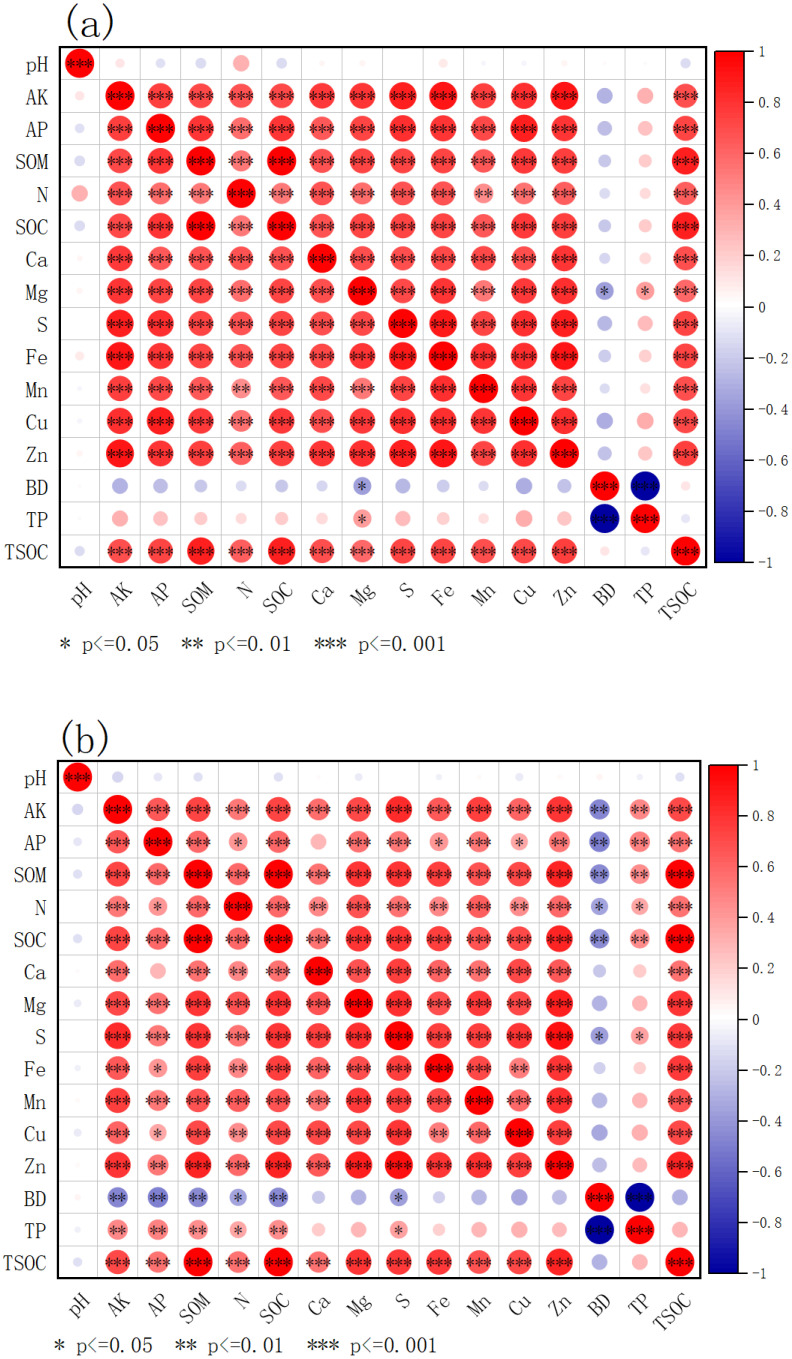
Correlation analysis diagram. **(a)** 0–20 cm surface soil; **(b)** 20–40 cm subsoil. AK, available potassium; AP, soil available phosphorus; SOM, soil organic matter; N, soil total nitrogen; SOC, soil organic carbon; pH, soil pH; Ca, exchangeable calcium; Mg, exchangeable magnesium; S, effective sulphur; Fe, effective iron; Mn, effective manganese; Cu, effective copper; Zn, effective zinc; BD, capacitance; TP, total porosity; and TSOC, soil organic carbon stocks.

A complex correlation between pH and most indicators such as available potassium, available phosphorus, total nitrogen, and organic carbon was observed (**Figure 3a**). It was found that available potassium was positively correlated with available phosphorus, total nitrogen, organic carbon. Organic carbon was positively correlated with trophic indexes such as exchangeable calcium, magnesium and sulfur. There was a significant negative correlation between bulk density and total porosity, as well as between bulk density and nutritional indexes such as available potassium and available phosphorus. Analysis of the topsoil layer (0–20 cm) revealed that different soil nutrients tend to enhance each other’s availability and with the enhanced compactness of soil (increased bulk density) the pore space got reduced which limits nutrient supply to plants. The interplay between nutrient synergism and soil compaction results in a unique ecological dynamic that influences soil fertility in this layer (**Figure 3a**).

A weak correlation between pH and nutritional indicators, such as available potassium and available phosphorus was found (**Figure 3b**). Available potassium was positively correlated with organic carbon, exchangeable calcium, and magnesium. Organic carbon was positively correlated with structural indexes such as exchangeable magnesium and available sulfur. A significant negative correlation between bulk density and porosity, and between bulk density and nutritional indexes such as available potassium and available phosphorus was also found. The correlation between certain trace elements (e.g., available manganese, available copper) and nutritional indicators shows significant differences compared to other elements (**Figure 3b**).

**Figure 4:**
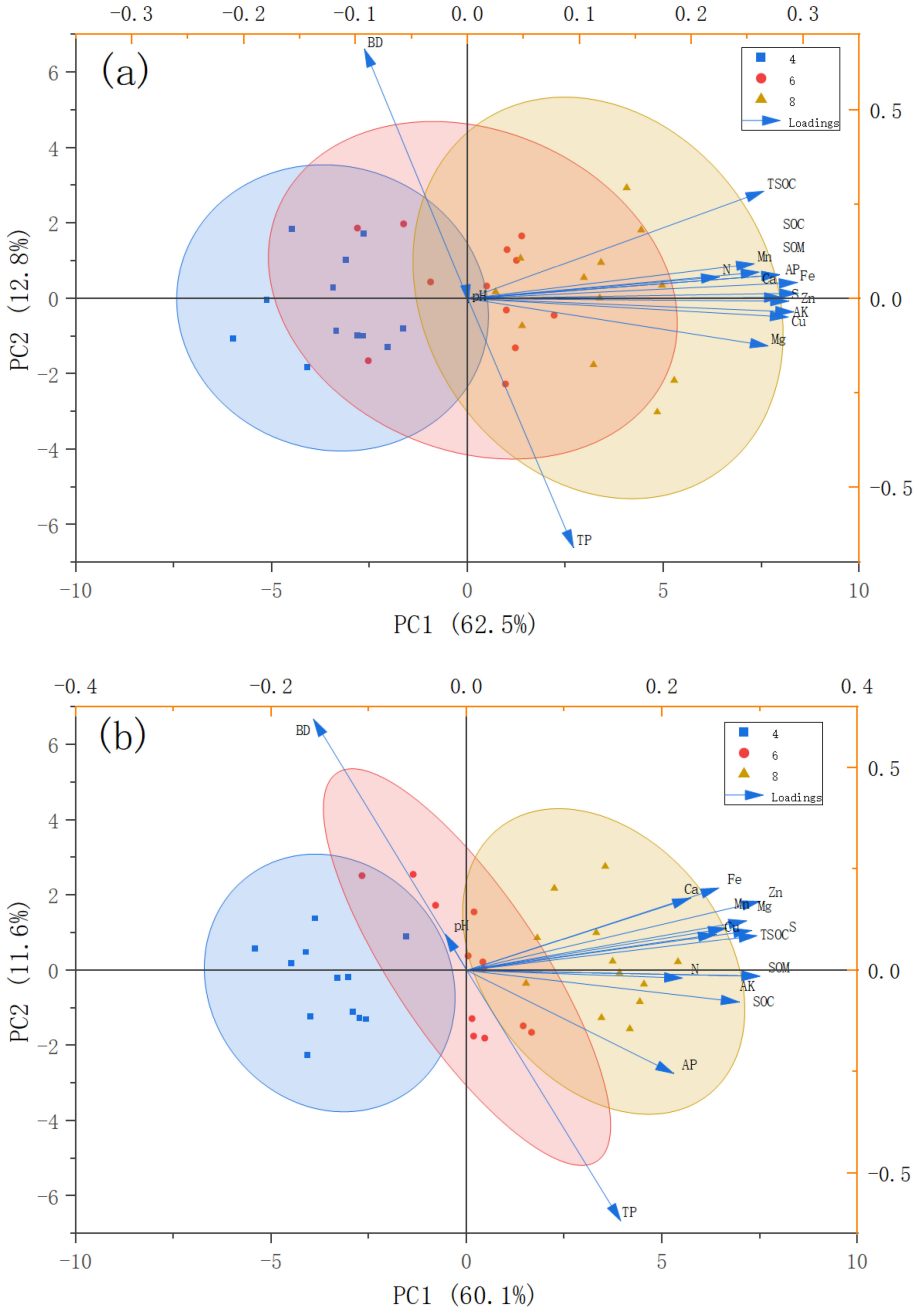
Combined PCA score and loading plot. **(a)** 0–20 cm surface soil; **(b)** 20–40 cm subsoil. AK, available potassium; AP, soil available phosphorus; SOM, soil organic matter; N, soil total nitrogen; SOC, soil organic carbon; pH, soil pH; Ca, exchangeable calcium; Mg, exchangeable magnesium; S, effective sulphur; Fe, effective iron; Mn, effective manganese; Cu, effective copper; Zn, effective zinc; BD, capacitance; TP, total porosity; and TSOC, soil organic carbon stocks. PC1 principal component 1; PC2, principal component 2.

For the 0–20 cm soil layer, the total explanatory rate of the soil layer for the mixed cropping pattern and soil physicochemical properties of pear grass was 75.3% (**Figure 4a**). According to the results of this PC analysis, it can be inferred that factors that contribute more to the difference of soil physical and chemical indexes are effective iron and effective zinc.

For the 20–40 cm soil layer, the total interpretation rate of different pear-grass mixed cropping patterns and soil physicochemical properties was 71.7% (**Figure 4b**). The PC analysis of soil layer indicated that factors that contribute more to the differences of soil physical and chemical indexes are effective zinc and effective sulfur.

Partial Least Squares Structural Equation Modeling (PLS-SEM) is a variance-based multivariate analysis method that employs iterative computations to maximize the predictive power of latent variables. This approach comprises two key components: the measurement model and the structural model, making it particularly suitable for exploratory research and analyzing complex causal mechanisms. Through evaluation metrics such as the coefficient of determination (R²) and the significance of path coefficients, PLS-SEM can effectively quantify inter-variable relationships and assess the model’s predictive performance.

**Figure 5:**
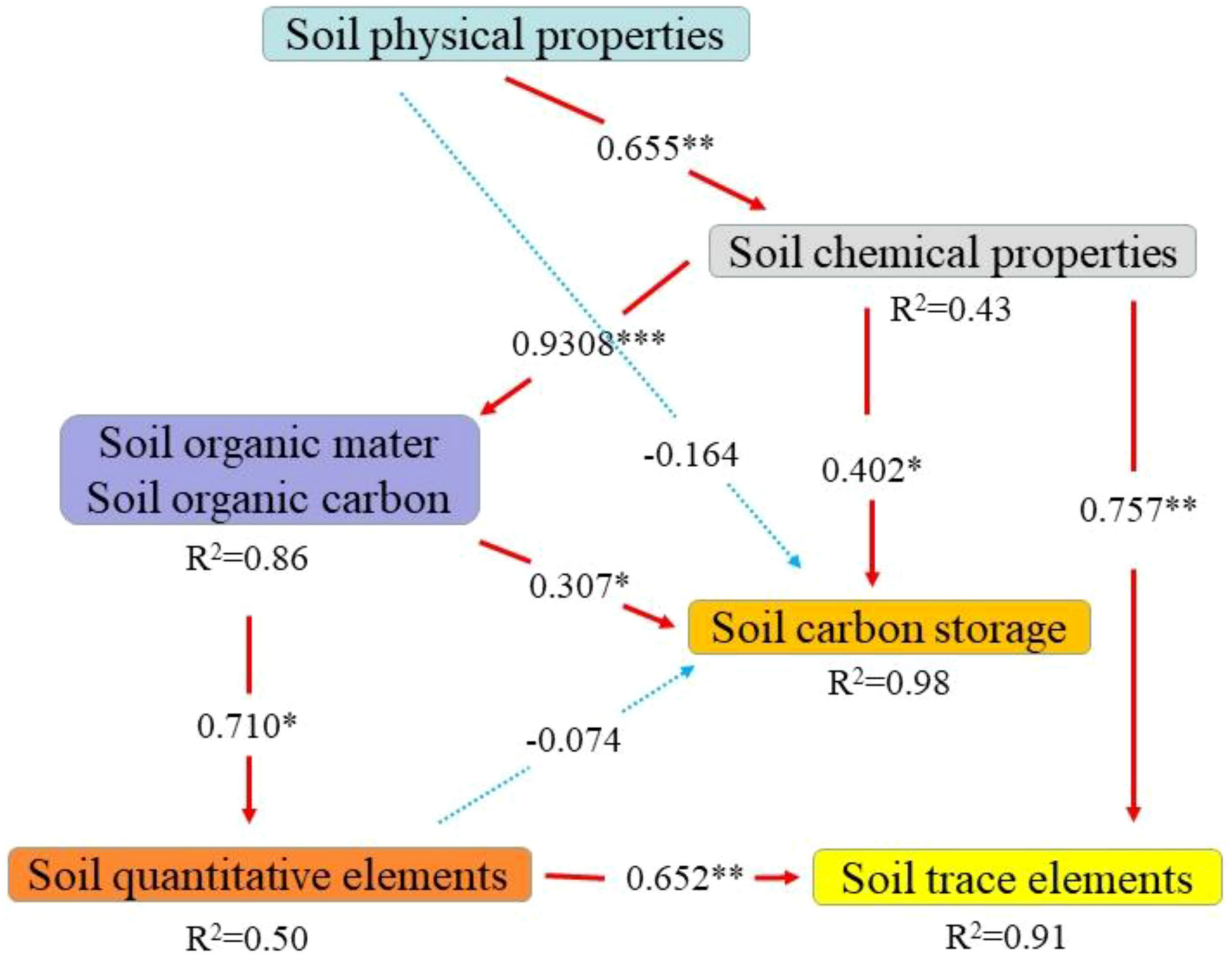
Pathway analysis of the impact of cover crop cultivation on soil carbon stocks. *, ** and *** indicate significant at *P<*0.05, *P<*0.01 and *P<*0.001 levels, respectively. AK-available potassium, AP-soil available phosphorus, SOM-soil organic matter, N-soil total nitrogen, SOC-soil organic carbon, pH-soil pH, Ca-exchangeable calcium, Mg-exchangeable magnesium, S-effective sulphur, Fe-effective iron, Mn-effective manganese, Cu-effective copper, Zn-effective zinc, BD-capacitance, TP-total porosity, and TSOC-soil organic carbon stocks. PC1 principal component 1, PC2- principal component 2. R^2^-coefficient of determination; red arrows indicate a negative effect, black arrows indicate a positive effect, and the thickness of the arrow line indicates the magnitude of the path coefficients in the model. Soil physical properties (AP, AK, TN), Soil chemical properties (TP, BD), Organic matter and organic carbon (SOC, SOM), Soil trace elements (Fe, Mn, Cu, Zn), Soil quantitative elements (Ca, Mg, S), Soil carbon storage (TSOC).

This study employed partial least squares structural equation model to analyze the influence mechanism of soil chemical properties, physical properties, organic matter, organic carbon, macro elements and trace elements on carbon storage. The GOF (goodness of fit) value of reached upto 0.8002, indicating that the fit was good. The results showed that the chemical properties of the soil were positively driven by the strong significant path coefficient of 0.655 (*P<*0.01). At the same time, a significant path coefficient of 0.402 (*P<*0.05) directly promotes the accumulation of carbon storage and participates in the carbon sequestration process. Organic matter and organic carbon served as primary components, with a highly significant path coefficient of 0.930 (*P<*0.001) indicating strong support for macro elements. Additionally, a significant indirect positive effect on trace elements was observed, with a path coefficient of 0.710 (*P<*0.01). It is noteworthy that physical indicators exert a negative effect of –0.1642 on carbon storage and an inhibitory effect of –0.079 on trace elements, indicating a potential ecological risk associated with changes in the physical state. Conversely, a strong positive correlation of 0.757 (*P* < 0.01) was observed between macro elements and trace elements, emphasizing their intermediary relationship (**Figure 5**).

## 4 Discussion

### 4.1 Land use modification as a driver of soil carbon storage enhancement

Land-use conversion in the ecologically vulnerable Taihang Mountain region significantly enhances soil carbon sequestration capacity, exhibiting pronounced spatiotemporal heterogeneity. Through quantitative analysis of ecological restoration project data from Fuping County, this study systematically reveals the enhancement mechanism of land use transition on carbon sink capacity. Statistical analysis demonstrates that orchard ecosystems exhibit significant carbon accumulation advantages over cropland systems (*P* < 0.05), with total carbon sequestration increasing by 43.4%. Dynamic monitoring of soil carbon pools shows that the soil carbon storage in 8-year-old pear orchards reaches 3.87 times that of traditional croplands, confirming the enhanced effect of perennial orchard systems on soil carbon sequestration. Efficiency evaluation further indicates that the carbon sequestration efficiency per unit area in pear orchards significantly surpasses that of cropland systems, highlighting the biological advantages of woody plant systems in carbon capture. Longitudinal comparative analysis reveals an orders-of-magnitude increase in regional carbon storage after transition compared to barren slopes and gneiss substrates (*P* < 0.01). These findings demonstrate, from four dimensions-system carbon storage, soil carbon pool capacity, carbon sequestration efficiency, and gneiss substrate transformation-the synergistic mechanism of orchard establishment in achieving carbon sink enhancement and ecological restoration in fragile ecological zones (**Table 2**).

This study systematically quantified the comprehensive effects of the “wasteland-to-orchard” model on ecosystem carbon sequestration. It organically integrated the vegetation-soil feedback mechanism proposed by Wang et al. (2024) with the canopy interception effect revealed by Tong et al. (2024), establishing a cascading feedback model of “canopy interception reducing erosion → soil environment stabilizing → litter input surging”. The observed 3-fold to 5-fold increase in litter input not only verified the correlation study by Zeng et al. (2024) but is also likely a key biological factor driving the continuous accumulation of soil active organic carbon. After 8 years of transformation practice, soil organic carbon increased by 2.3-4.1 g/kg, and microbial biomass carbon rose by 58%-76%, confirming the long-term feasibility of the ecological restoration pathway proposed by Owusu et al. (2024) from a temporal perspective. However, scientific issues such as the specific responses of different tree species in the feedback cycle, the dynamics of inert carbon transformation, and the impact of extreme climates on carbon sequestration still require in-depth exploration. Future research should combine molecular biological techniques with long-term *in-situ* observations to systematically analyze the carbon cycle mechanisms in the “plant-soil-microbe” system, providing more precise theoretical support for regional ecological restoration.

### 4.2 Carbon input and sequestration in pear-cover crops strategy

This study systematically quantified the effects of cover crops on soil organic carbon in pear orchards, revealing a novel mechanism through which herbaceous plants regulate the vertical distribution of soil carbon pools via root system architecture. Contrary to conventional understanding that primarily emphasizes litter input (Smith et al., 2020), we found that deep-rooted herbaceous plants (e.g., winter rapeseed) significantly enhanced soil organic carbon in the 20–40 cm deep layer (with an increase of 22.43% in 8-year-old orchards), surpassing the previous limited focus on surface carbon pools. In the unique shallow soil-bedrock abrupt-interface habitat of Fuping, deep-rooted plants successfully activated the traditionally difficult-to-utilize deep carbon pool, providing new insights into carbon cycle research in fragile ecosystems. While this study validates the theory that deep-rooted plants promote subsoil carbon sequestration (Borchard et al., 2019), it further reveals differences in carbon sequestration efficiency among herbaceous species: ryegrass demonstrated optimal performance in the surface layer (0–20 cm), with an increase of 18.51%-24.43%, whereas winter rapeseed exhibited greater advantages in deeper layers. This species-specific variability highlights the necessity of establishing a multidimensional evaluation system.

The positive correlation between tree age and carbon sequestration capacity observed in this study is consistent with findings from He et al. (2023) and Li et al. (2023a). However, it remains unclear whether this trend plateaus as trees mature and how management practices specifically regulate this mechanism. This experiment confirms that winter rapeseed exhibits better carbon sequestration performance than ryegrass in the 20–40 cm subsoil layer. This phenomenon can be reasonably explained by root architecture theory: the advantage of deep-rooted plants (e.g., winter rapeseed) over fibrous-rooted plants primarily stems from their efficient utilization of the vertical spatial niche, which is realized through two key mechanisms. On the one hand, deep root systems penetrate compacted subsoil, and during growth, root deformation and soil compression, along with the secretion of organic substances, directly promote the formation of large aggregates (Kell, 2011), providing a physical protection barrier for organic matter and thereby enhancing carbon stability. On the other hand, deep roots serve as the main pathway for transporting organic carbon into the subsoil. The root-derived carbon input, due to its complex chemical composition and the slow-decomposition environment of deeper soil layers, exhibits a longer turnover time (Rasse et al., 2005). The activation of the deep soil carbon pool observed in this study strongly supports the synergistic effect of these two mechanisms. Although the theory proposed by Lynch (1995) provides foundational support for root penetration into the subsoil, the more recent studies cited above offer more precise insights into the underlying mechanisms. Nevertheless, the relative contributions of physical protection versus biochemical pathways to carbon pool stability require further clarification using techniques such as isotope labeling.

### 4.3 Soil optimization and carbon storage in pear-cover crops approach

This study confirms that the pear-herb composite system significantly optimizes soil physical structure by reducing bulk density and increasing porosity, a finding that aligns with the emphasized role of plant root systems and organic matter in improving soil structure, as noted by Blanco-Canqui and Ruis (2020). More importantly, our research quantitatively reveals a negative correlation between bulk density and soil organic carbon content through structural equation modeling, thereby clarifying that physical structure optimization is a key pathway driving soil organic carbon preservation. This provides mechanistic support for the reduced carbon footprint observed in intercropping systems by Zhang et al. (2023). However, the carbon sequestration effects of improved soil structure may have certain boundaries. This study observed significant vertical heterogeneity in the improvement effects within the soil profile, primarily due to the vertical decline in herb root distribution and organic matter inputs. This new insight suggests that future assessments of carbon sequestration effects must more carefully consider the influence of soil depth. Furthermore, although the contribution of organic matter inputs to carbon sequestration is significant, its long-term stability, dynamics under varying climatic conditions, and potential saturation points remain uncertainties that require further quantification.

Several research gaps remain. For instance, how differences in root architecture and exudates among different herb species and their combinations specifically influence the formation and stability of soil aggregates warrants deeper investigation (Ogilvie et al., 2021). Additionally, when extending this pear-herb model to broader fragile ecosystems, a comprehensive assessment of its long-term ecological benefits and economic feasibility will be a critical direction for future research. Despite these uncertainties, the synergistic model of “structural improvement-carbon sequestration enhancement” established in this study provides a solid theoretical and practical foundation for enhancing ecosystem services through optimized agroforestry systems (Lal, 2018), highlighting the significant potential of soil management in addressing global change.

### 4.4 Nutrient-carbon interactions in pear and cover crops systems

This study elucidates the mechanism through which herbaceous intercropping systems enhance soil carbon pool stability via the dual pathways of “nutrient-driven accumulation” and “alkalinity-constrained decomposition”. While this qualitative framework aligns with the functional model of cover crops proposed by Cong et al. (2015), our research provides the first quantitative evidence of the central role of soil chemical properties (R²=0.43) in driving carbon storage, a novel finding that challenges the conventional emphasis on physical factors and offers a new perspective for understanding the carbon sink function of intercropping systems. This study reveals a dual-pathway mechanism for nutrient-carbon pool synergy: (1) In the biochemical pathway, simultaneous increases in available potassium and phosphorus alleviate microbial nutrient limitation, converting organic residues into stable microbial-derived carbon through the “microbial carbon pump” (Liang et al., 2017); (2) In the physicochemical pathway, potassium ions promote soil colloid flocculation and aggregate formation, while available phosphorus complexes with iron-aluminum oxides to jointly provide physical protection for organic carbon (Kleber et al., 2015). PLS-SEM path analysis supports this mechanism (path coefficient = 0.402), confirming that enhanced nutrient availability achieves carbon sequestration through dual pathways of “promoting transformation and inhibiting decomposition”. Specifically, the observed increase in available potassium and phosphorus (17.26%-25.45%) within the intercropping system and its positive correlation with total organic carbon corroborates, from a plant nutrition-driven perspective, the findings of Fu et al. (2023) in tropical soils. More significantly, this study systematically reports, for the first time, the quantified contribution of alkaline cations (exchangeable Ca/Mg increased by 16.58%-25.46%) in suppressing carbon decomposition. This provides direct empirical support for the theory of “base environment promoting organic carbon stability” proposed by Rodrigues et al. (2021). Notably, the structural equation model revealed a negative impact of physical indicators on carbon storage. This unexpected finding suggests the potential existence of unrecognized compensatory mechanisms within the system. A significant knowledge gap remains regarding the specific pathways of the trace element network (R²=0.91)—particularly the relative contributions and interactions of different trace elements—which constitutes a critical direction for future research. Although this study establishes a coupled chemical-physical-biogeochemical mechanism, its universality across different soil types and climatic conditions requires broader validation.

These uncertainties indicate that the complex systems theory proposed by Figueiredo et al. (2025) necessitates a more refined assessment framework. Future research should focus on: (1) the species-specific effects of different herbaceous plant combinations on alkaline cation composition; (2) the underlying mechanisms for the negative effects of physical indicators; (3) the threshold effects of the trace element network in carbon stabilization. Despite these challenges, the integrated model established in this study undoubtedly opens new avenues for enhancing ecosystem carbon sink functions through the precise management of soil chemical properties.

## 5 Conclusions and prospects

This study presents a typical example of Nature-based Solutions through ecological restoration practices implemented in the fragile ecological region of gneiss slopes in the Taihang Mountains. The transformation of barren slopes into pear orchards combined with herbaceous plant intercropping has successfully achieved multiple synergistic benefits, including enhanced soil carbon sequestration capacity and improved ecosystem services, embodying the core concept of “addressing climate change synergistically and enhancing human well-being through sustainable ecosystem management”. Specifically, as pear orchard stands age, soil carbon storage demonstrates a significant accumulation trend, showing notably greater carbon sequestration potential compared to traditional cropping systems. Furthermore, intercropping with deep-rooted herbaceous plants not only effectively promotes carbon storage in both surface and subsurface soil layers but also improves the physical structure and nutrient status of rocky soils through root penetration. This technical pathway of optimizing soil three-phase structure (reducing bulk density, increasing porosity) and establishing nutrient-carbon pool synergy fully demonstrates the technical characteristics of “harnessing natural processes to achieve multiple synergistic objectives”.

Future research should focus on developing a pear-herb combination selection system adapted to the ecological characteristics of gneiss hilly areas. Key priorities include: analyzing the regulatory mechanisms of different herbaceous plants on soil labile and stable carbon fractions, establishing a compatibility assessment framework that integrates topographic parameters and soil factors, and investigating spatial interaction patterns between woody and herbaceous root systems. Ultimately, this will lead to optimized configurations that balance carbon sequestration effectiveness with ecological adaptability. Advancement in these research directions will provide theoretical support for precision restoration of degraded soils in ecologically fragile areas, thereby enriching the practical implications of Nature-based Solutions.

## Data Availability

The raw data supporting the conclusions of this article will be made available by the authors, without undue reservation.
